# Assessing the stability of indoor farming systems using data outlier detection

**DOI:** 10.3389/fpls.2024.1270544

**Published:** 2025-03-12

**Authors:** Jean Pompeo, Ziwen Yu, Chi Zhang, Songzi Wu, Ying Zhang, Celina Gomez, Melanie Correll

**Affiliations:** ^1^ Department of Agricultural and Biological Engineering, University of Florida, Gainesville, FL, United States; ^2^ Department of Horticulture and Landscape Architecture, Purdue University, West Lafayette, IN, United States

**Keywords:** Arduino, low-cost sensor, outlier, uncertainty, decomposition, regression

## Abstract

**Introduction:**

This study investigates the quality of air temperature data collected from a small-scale Controlled Environment Agriculture (CEA) system using low-cost IoT sensors during lettuce cultivation at four different temperatures. Ensuring data quality in CEA systems is essential, as it affects system stability and operational efficiency. This research aims to assess system stability by analyzing the correlation between cumulative agricultural operations (Agr.Ops) and air temperature data variability.

**Methods:**

The methodology involved collecting air temperature data from IoT sensors in the CEA system throughout lettuce cultivation trials. A generalized linear model regression analysis was conducted to examine the relationship between cumulative Agr.Ops and the z-scores of air temperature residuals, which served as an indicator of system stability. Outliers in the sensor data were identified and analyzed to evaluate their impact on system performance. Residual distribution and curve fitting techniques were used to determine the best distribution model for the sensor data, with a log-normal distribution found to be the best fit.

**Results:**

Regression analysis indicated a strong inverse relationship between cumulative Agr.Ops and residual z-scores, suggesting that increased Agr.Ops correlated with a higher presence of outliers and a decrease in system stability. The residual analysis highlighted that outliers could be attributed to potential issues such as sensor noise, drift, or other sources of uncertainty in data collection. Across different trials, the system displayed varying degrees of resistance to cumulative Agr.Ops, with some trials showing increased resilience over time.

**Discussion:**

The alternative decomposition method used effectively identified outliers and provided valuable insights into the functionality of the system under different operational loads. This study highlights the importance of addressing uncertainties in indoor farming systems by improving surrogate data models, refining sensor selection, and ensuring data redundancy. The proposed method offers a promising approach for enhancing monitoring and managing uncertainties in CEA systems, contributing to improved stability and efficiency in indoor farming.

## 1 Introduction

By 2050, the world’s population is expected to reach 9.7 billion (United Nations and Department of Economic and Social Affairs and Population Division, 2019: Highlights, 2019), and roughly two-thirds of this population are projected to be living in cities (Ramin Shamshiri et al., 2018). To meet the food requirements of this increasing population, the global food system will be forced to increase food production on a dwindling supply of agricultural land (Plaut, 1980). In addition to this, extreme and increasingly irregular weather events (Wehner et al., 2017) and clean water scarcities (Boretti and Rosa, 2019) are expected to further threaten the resilience of traditional agricultural and food production systems. Although challenging, innovative approaches to agricultural production are needed to ensure global food security (Keating et al., 2014; Lipper et al., 2014) and adequate food supplies under these conditions (Pitesky et al., 2014). Controlled environment agriculture (CEA) production methods like greenhouses and vertical farms can help meet the challenge of more intensive, profitable and sustainable production (O’Sullivan et al., 2019; Coon et al., 2024). CEA allow for year-round crop production, including in areas where crops otherwise could not be grown (Jensen and Malter, 1995). The greenhouse production of specialty crops (fruits, vegetables, and floriculture) is an important part of U.S. agriculture, with a $6.9 billion in annual wholesale farmgate value (USDA et al., 2019). Compared to field production, greenhouses generate higher yields per unit growing area, all while using less water, fertilizer and pesticides per unit yield (USDA et al., 2020). CEA systems are potentially viable strategies for mitigating the many complex challenges affecting global food production (Ramin Shamshiri et al., 2018; Iddio et al., 2020; Engler and Krarti, 2021). CEA systems can improve water and nutrient use efficiencies, provide healthier and safer foods with little to no chemical pesticide applications, increase yields per square foot of land, reduce transportation costs when in urban settings, and provide consistent high-quality food production year-round despite climatic conditions. However, some of the main disadvantages of CEA include high capital investment and operating costs caused by high energy consumption and labor requirements (Benke and Tomkins, 2017; Engler and Krarti, 2021; Van Delden et al., 2021) which limit their financial profitability (O’Sullivan et al., 2019). In order to use CEA to improve the sustainability and resiliency of our food production systems, it is imperative to improve their economic viability (Hati and Singh, 2021) and accessibility.

Two of the main types of CEA systems include greenhouses and vertical farms, each with specific benefits, drawbacks, and optimal use cases. Greenhouses consist of semi-controlled environments where plants can be grown in soil or hydroponically, with controlled irrigation and fertigation, and primarily using sunlight for lighting and heating requirements. These semi-controlled environments can improve plant yield and quality, reduce pest and disease pressures, and extend growing seasons for crops when compared to conventional production methods. For example, lettuce grown hydroponically in a greenhouse used 13 ± 2.7 times less water and yielded 11 ± 1.7 times more than conventionally grown lettuce, though it did require 82 ± 11 times more energy (Barbosa et al., 2015). Vertical farms grow plants with soilless cultivation methods in air-tight, thermally insulated buildings with controlled lighting and environmental conditions which can provide great benefits when compared to greenhouses. A comparison of plant factories (a type of vertical farm) and greenhouses found that plant factories outperformed even the most efficient greenhouses, achieving higher water, carbon dioxide, and land area productivities (Graamans et al., 2018). One vertical farm in Japan produced 100 times more lettuce per square foot with 40% less energy, 80% less food waste, and 99% less water uses than a conventional farm (Hati and Singh, 2021).

In CEA, temperature sensors are used for environmental monitoring and control systems (heating, ventilation, air conditioning, shade structures, dehumidifiers, humidifiers, irrigation, etc.) to achieve precise control of the environment around plants. Temperature can have a significant impact on the resource use efficiencies of the system by affecting heating and cooling loads, water use rates, sensor readings, and even the lifespan and energy conversion efficiencies of components making up the system. Multiple plant growth and development factors are affected by temperature, including photosynthesis, respiration, growth rate, developmental stage, germination, and plant height (Yang et al., 2014), with optimal ranges varying between plant species and cultivars. Hot temperatures above 35°C have been shown to harm the photosynthetic process (He et al., 2009), and having temperatures that promote plant respiration at night while reducing photosynthesis rates during the day can reduce overall plant yield (Yamori et al., 2014). Root-zone temperatures affect metabolic enzyme activity and the rate of water uptake in plants (He et al., 2009), with warmer temperatures leading to faster growth rates and greater water use. Root-zone temperatures also affect the dissolved oxygen (DO) concentration in water, with warmer temperatures reducing oxygen concentrations which can lead to plant death when oxygen concentrations become too low (Brechner et al., 1996). The electrical conductivity (EC) readings of nutrient solutions used for hydroponic production need to be compensated for the temperature to ensure accuracy since EC has a direct relationship with temperature. LEDs used for artificial lighting become less energy efficient when temperatures exceed their optimal ranges, eventually leading to a decrease in optimal efficiency and more heat generation (Peng and Liu, 2021). Furthermore, external operating temperatures and the different types of CEA systems used can lead to different optimal conditions for energy efficiency, with warmer setpoints in hot conditions and cooler setpoints in cold conditions often being more efficient (Yang et al., 2014; Zhang and Kacira, 2020). Thus, precise temperature control is essential for the effective and efficient operation of CEA production systems.

Sensors are a crucial component for monitoring and controlling environmental conditions inside CEA systems to ensure accurate conditions are maintained. Using sensors can optimize resource use efficiencies, reduce labor requirements, and ensure the production of high quality crops. Low-cost (LC) sensors provide affordable and scalable ways of establishing the dense sensor matrices that are necessary for accurately monitoring and controlling CEA operations. Internet of Things (IoT) systems use LC sensors in a variety of ways to improve agricultural activities without the hefty price tag associated with high quality sensors. IoT devices are enhanced with computational and networking power often through open-source software (Charumathi et al., 2017; Tzounis et al., 2017; Marques et al., 2019; Montoya et al., 2020; Tatas et al., 2021), and using multiple devices allows for the collection of large quantities of data. With larger amount of data, various system operations or incidences can be interpreted or represented through proper analysis methods, such as statistics. Some of these operations or incidences include sensor calibration (Montoya et al., 2020), intermittent loss of connection, lack of data trustworthiness and thus poor decision making (Karkouch et al., 2016), unstable and congested networks, and the exhaustion of power supplies (Teh et al., 2020). These issues are prone to data quality cases such as outliers, missing data, bias, drift, noise, and uncertainty (Teh et al., 2020).

Solutions for interpreting data quality information include a variety of sophisticated data processing techniques such as principal component analysis (PCA), artificial neural networks (ANN), and Bayesian Networks, that enhance the functionality and usability of these systems by addressing those errors (Teh et al., 2020; Samara et al., 2022). For time series data, statistical and machine learning methods have been used for detecting anomaly data, such as outliers (Filimonov et al., 2020; Fawzy et al., 2013). Staged rules (e.g., physical limitation test, step test, internal identity test, etc.) were developed for meteorology and weather data monitoring (Tak, 2015; Cella et al., 2019; Kim et al., 2015; Oh et al., 2013). In agriculture, and especially controlled environment operations, data quality analysis is critical for the management of environmental systems related to power consumption, crop growth, productivity, system stability, etc. However, related studies in the agriculture field within relevant data sets are few, if any.

In this study, the quality of air temperature data collected from a small scale CEA system using low cost IoT sensors is investigated to assess the stability of the system during the cultivation of lettuce at four different air temperatures. The system configuration and the methodology of analysis will be introduced next, followed by the results of statistical analysis. The findings and insights will be provided in the discussion, followed by the conclusion.

## 2 Materials and methods

In this experiment, time series data was collected from various environmental sensors connected to an Arduino based sensor array within a vertical farming (VF) system where nutrient film technique (NFT) hydroponic lettuce was grown under LED lights. The sensor array output data to a MySQL database and logged data locally to a computer throughout the experiment as a backup data set (See details in the Appendix). The controlled nature of this small scale VF made for regular temporal fluctuations that were easily detectable and based primarily on the functionality of the system (equipment, timers, sensors, etc.). External environmental conditions had negligible effects on the VF since it was insulated within a building with constant environmental control. The small size of the VF made the internal environmental conditions prone to a high degree of fluctuation caused agricultural operations, and thus a potentially influential factor on the system functionality and uncertainties in the environmental data. Air temperature data was used to apply statistical techniques for residual analysis and outlier detection of the decomposed time series data as a measure of the stability of the system. The data analysis method proposed in this experiment could help to reduce the overall operating costs of vertical farms by improving energy use efficiencies and reducing labor requirements.

### 2.1 Environment overview

An environmental growth chamber was used to house an indoor farm system for this study with experiments involving lettuce growth. The chamber’s temperature, humidity, and airflow were controlled, and CO2 was enriched to a constant level. The experiment involved four trials with different air temperature set points. Data was collected using six microcontroller boards connected to sensors for various parameters such as air temperature, humidity, CO2 concentration, and nutrient solution conditions. The data was logged online to a MySQL database and locally as a.txt file. Sensors were placed at different locations in the chamber to monitor conditions (See details in the Appendix). The experiment aimed to study the growth and development of lettuce under different environmental conditions. The data management system included a database design to store real-time data and manage sensor devices, as well as a backup file system for data security. The details information about the system design can be found in the appendix.

### 2.2 Data in this study

This study focuses on the air temperature data collected from the chamber. The air temperature was controlled by the air conditioning system of the building to ensure it uniformity and monitored at two plant canopy levels and other locations to record its variations. The experiment involved four trials labelled: T1, T2, T3, and T4, each with a different day-time air temperature set point of 30°C, 24°C, 28°C, and 26°C respectively. Data was collected using five LC air temperature sensors (Adafruit, SHT30, New York, NY) connected to microcontroller boards, with readings taken at 10-second intervals, as well as an independent data-logging reference air temperature sensor (Hydrofarm, APCEM2, Petaluma, CA) (See details in the Appendix). The collected data included 302,400 data points per trial.

Sensors were calibrated and placed at various locations to monitor the air temperature inside the chamber accurately and understand temperature uniformity at different points. A low-cost sensor was placed at each of the two plant canopies (LC_Canopy_1, LC_Canopy_2) to monitor them for uniformity. A LC sensor was installed next to the reference sensor in an open space in the chamber to monitor overall environmental conditions in the chamber and to compare the accuracy of readings between the two at the same location. Two other temperature sensors were placed at the intake of the AC to monitor energy use of the unit, and another outside the chamber to monitor ambient conditions of the building housing the chamber. Of the six sets of air temperature data collected, only four were used for analysis: the reference sensor (Reference), and three LC sensors located at both plant canopies and next to the reference sensor (LC_Canopy_1, LC_Canopy_2, LC_Reference). The other two temperature sensor data were not considered in our analysis due to time constraints and relevance to the study. Other environmental data such as relative humidity and CO2 concentrations were outside the scope of this study due to budgetary and time constraints, a lack of reference sensor data, and because these data displayed irregular seasonality that would require a different analysis method than what was proposed in this study (see details in the appendix). All sensors were connected to the same power supply, so electrical fluctuations should have affected them all similarly.

Agricultural operations were monitored and logged as two data points, 1) door status, 2) person status. Both were logged as binary variables indicating open/closed for the door status, and present/absent for person status. Door status was recorded automatically using a photocell (Adafruit, CdS photocell, New York, NY) that was attached to the chamber door which would detect light when the door was open (1), and no light when the door was closed (0). Person status was manually recorded using a switch state, three button-based logging system, where one would press one of the three buttons to record being in the chamber and change its state to “present” (1), and then press the button again when leaving the chamber to change its state to “absent” (0). This allowed us to record the simultaneous presence of up to three people in the chamber which would have a three-fold effect on the environmental conditions as that of a single individual working in the chamber.

### 2.3 Data classification method

All data processing and analysis was performed with R Statistical Software (RRID:SCR_001905) (R Core Team, 2024). Multiple libraries were used for data wrangling and processing including ‘lubridate’ (Grolemund and Wickham, 2011), ‘tidyverse’ (Wickham et al., 2019), ‘plyr’ (Wickham, 2011), ‘dplyr’ (Wickham et al., 2023), ‘ggplot’ (Wickham, 2016), ‘ggpubr’ (Kassambara, 2023), data.table (Barrett et al., 2024), ‘psych’ (Revelle, 2024), ‘hrbrthemes’ (Rudis, 2024), ‘devtools’ (Wickham et al., 2022), ‘fitdistrplus’. Decomposition was performed using the base R stats package. Regression analyses were performed with base R stats package using the generalized linear model (glm) function.

#### 2.3.1 Missing data processing

**Figure 1:**
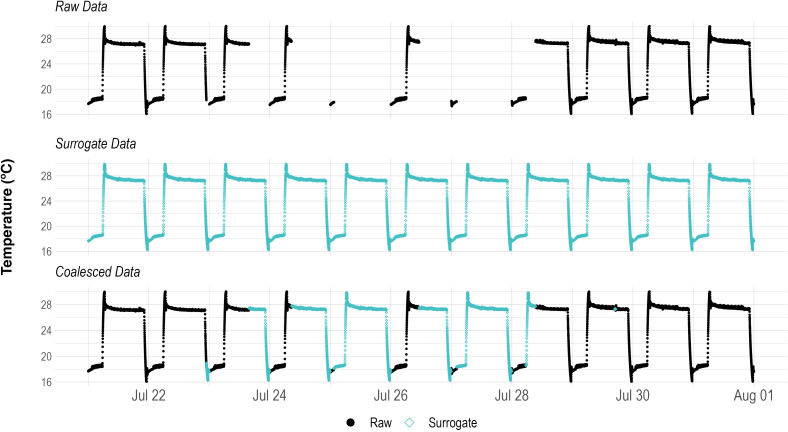
Replacing missing data points in the raw sensor data with surrogate data values that match the hour/minute time signatures. The resulting coalesced data set has no missing data values and is used for decomposition.

The data collected throughout the trials had many missing data points with varied causes such as sensor and microcontroller malfunctions, power surges, and power outages. The missing sensor data points were replaced with data from a surrogate data set that matched the date time timestamp per sensor and per trial (**Figure 1**). The resulting data set was free of missing data points and is referred to as the coalesced data throughout this document. The surrogate data sets that were used consisted of the median sensor reading per trial for every unique hour/minute combination during a 24 hour time span. The median value was selected as it is less distorted by outliers than the mean and is as such a more appropriate measure of central tendency (Khorana et al., 2023) per data point. These median values resulted in complete 24 h data sets that more accurately identified the temperature seasonality per sensor and per trial when compared to a standard additive decomposition function.

#### 2.3.2 Data decomposition

**Figure 2:**
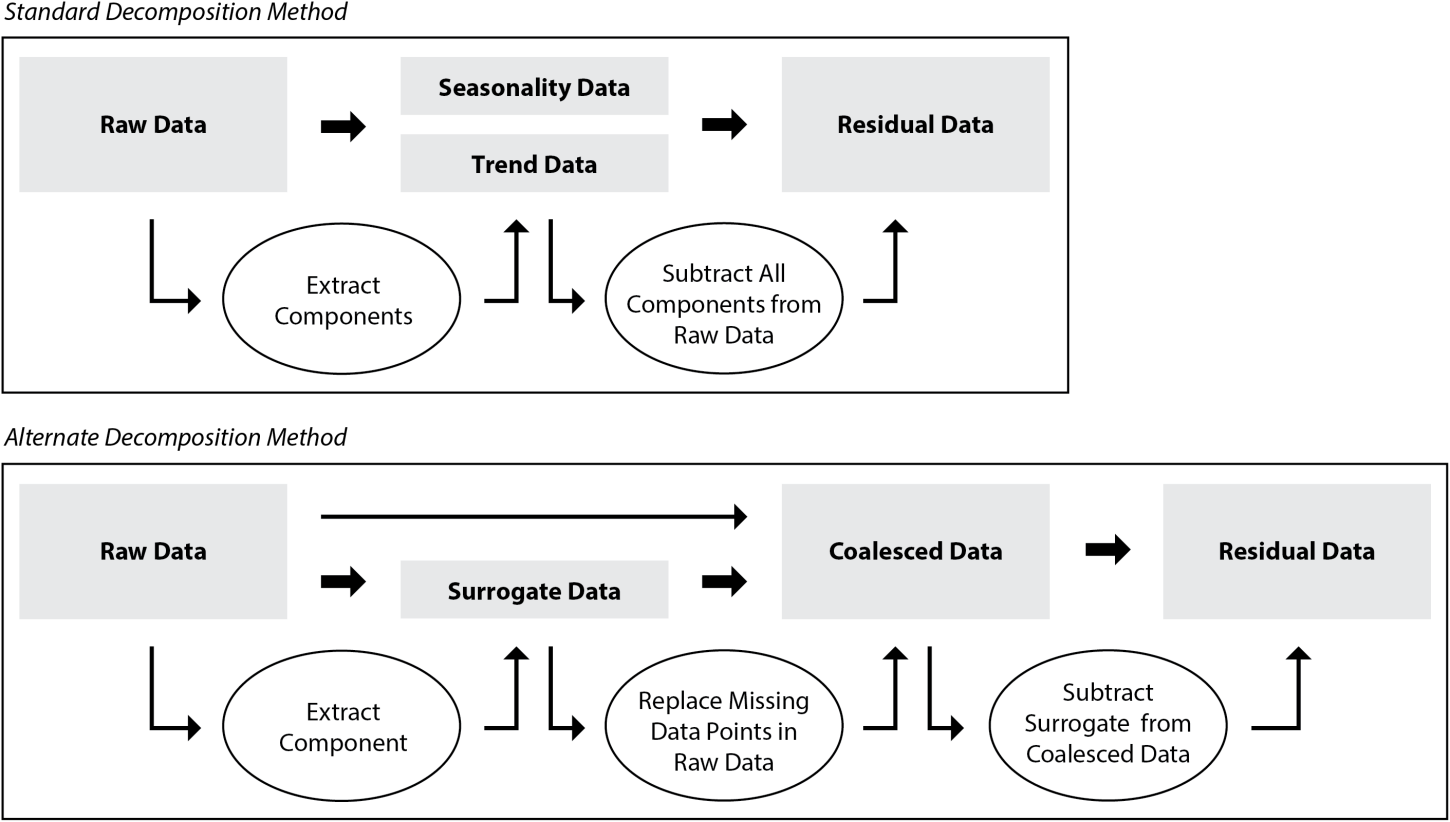
Standard decomposition method (above), and the alternate decomposition method (below) proposed in this experiment that was used for decomposition of air temperature data.

Time series data can be processed in a number of ways in order to extract more meaningful statistics and other such characteristics of the data. One such method is decomposition, which is able to separate time series data into its seasonal and trend pattern. By subtracting the extracted patterns from the original data set, one can look at the noise, or residuals, within the data. One type of decomposition, called additive decomposition, is effective when the peaks present in the seasonality of the data do not vary much over time (Prema and Rao, 2015), such as the data collected from an indoor farming system where temperature fluctuations are regular and controlled, and are little influenced by external conditions. However, this method fails when there are large amounts of missing data, as was the case during this experiment. The missing data issue was remedied using the surrogate data sets to fill in missing values and generate coalesced data sets. Inputting the coalesced data through a standard decomposition function yielded seasonality and trend components, which could be subtracted from the coalesced data to identify the residuals. However, the surrogate data essentially represented the periodicity of the raw data, so by subtracting the surrogate from the coalesced data, one could effectively de-seasonalize it (Prema and Rao, 2015) and output the residual data used for outlier identification (**Figure 2**) (Blázquez-García et al., 2022). This alternative decomposition method proved more successful at extracting the residual values than employing a standard additive decomposition function. Thus, the residual data sets of the alternative decomposition method were used for residual analysis.

In the air temperature data, visible daily periodicities caused by day/night setpoints matched the requirements of using an additive decomposition method. These daily temperature fluctuations did not vary greatly within an individual temperature trial, however the periodicities differed between the different trials due to the four distinct day temperature set points used and as such, the air temperature data from each trial was decomposed separately. Furthermore, each temperature sensors unique data sets were decomposed separately to study the performance of the individual sensors. Cumulative agricultural operations data were used to identify existing correlations between the number of outliers and the stability of the system over time.

#### 2.3.3 Residual calculation and outlier detection

The seasonality and trend data of larger and more complete data sets can be more successfully extracted when compared to smaller and more flawed data sets. Thus, smaller and more flawed data sets generally lead to lower quality residual extraction which are then less usable for analysis or diagnosis. The “surrogate data” as proposed in this study represents the median daily oscillating pattern of a system, and can be used to replace missing values in its parent data set. However, much like with standard decomposition, its uses are limited by the quantity and quality of data collected since insufficient data or an unstable sensor would yield surrogate data that cannot effectively fill missing data or be used for decomposition. As the data set increases in size, so does the reliability and usability of the surrogate data for system diagnosis.

**Figure 3:**
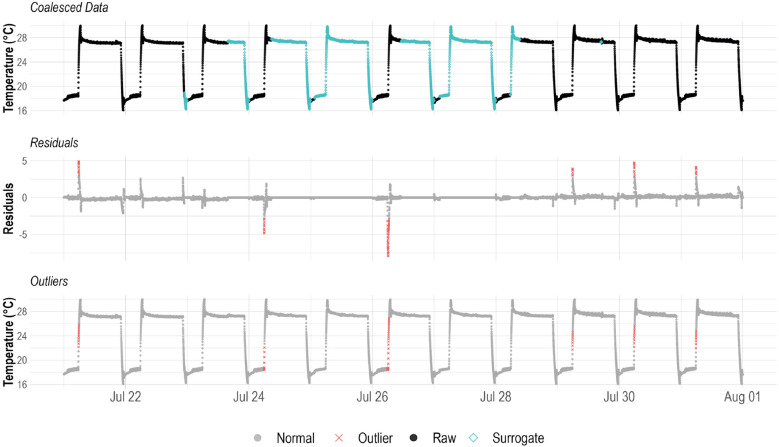
A visualization of the alternate decomposition method for the coalesced data from the LC sensor into residuals with identified outlier (|z| > 3.29) data points. The identified outlier data are then displayed in the coalesced data to better demonstrate their temporal relevance.

Residual distributions were analyzed for spread, central tendency, and normality as a measure of data and sensor quality (Montoya et al., 2020). Assuming manufactural calibration of sensor was successful, the residuals should be normally distributed with a mean trending towards zero. Statistical analysis such as z-score tests have been performed on residuals with a significance level of α = 0.05 (Montoya et al., 2020), however, a smaller value of α = 0.001 was used in this experiment after testing this value with the data from previous trials and finding that it better demonstrated results. Residual data with z-scores greater than 3.29 standard deviation from the mean (|z| > 3.29) were labelled as outliers (**Figure 3**). By studying the residual data, changes in the regular oscillations of the system can be analyzed to identify uncertainties, outliers, sensor errors, and identify which operations are critical and how they impact the stability of the indoor farming operation (**Table 1**). The distribution of the absolute value of the z-score data was analyzed and to identify the best fit curve to this distribution and to inform the regression analysis.

**Table 1:** Uncertainties of the indoor farming system.

Types of Uncertainties	Controllable?	Potential Impact	Possible Solution
Power Outage	No	Data collection, system operation, missing data	Backup power supply
Power Surge	No	System operation, sensor damage, data corruption, missing data	Surge protectors
Malfunction – AC Condenser	No	Status of the system	Not Applicable
Malfunction – AC Fans	No	Status of the system	Not Applicable
Malfunction – AC Temperature Sensor	No	Status of the system, sensor damage	Not Applicable
Malfunction – AC Humidity Sensor	No	Status of the system, sensor damage	Not Applicable
Malfunction – Arduino board	No	Data collection, sensor damage, data corruption, missing data	Check regularly
Malfunction – Python code	No	Data collection, data corruption, missing data	Warning message
Malfunction – Float sensor	No	Status of system, sensor damage, missing data	Check regularly
Human Error	Yes	Data collection, status of system, sensor damage, missing data	Not Applicable
Light Intensity Reduction	No	Status of system	Not Applicable
Chamber door opening/closing	Yes	Status of system	Lock door
Agricultural operation duration	Yes	Status of system	Limit tasks to specific time intervals
Internet Outage	No	Data collection, missing data	Not Applicable
Sensor Drift	No	Status of system	Regular calibration
Biofilm Accumulation	No	Status of system, sensor damage	Regular maintenance and sterilization

#### 2.3.4 Regression analysis

A generalized linear model was used to perform a regression analysis between the cumulative agriculture operations (Agr.Ops) and the corresponding z-scores of air temperature residual data to assess the overall stability of the operating system. Agr.Ops were quantified cumulatively per trial to account for the progressive effect of the system stability. If Agr.Ops impacts system stability, as the cumulative number of Agr.Ops increased over time, we expected to see an increase in the number of outliers. Our hypothesis was that the amount and the intensity of data outliers can inform the stability of the system operation and its correlation with Agr.Ops. However, to study this correlation, we needed to remove all non-outlier values from the analysis as they made up more 97% of the data and would have skewed the results. Thus, finding the best fit curve for the distribution and regression analysis were both performed with the outlier data only.

## 3 Results

### 3.1 Data summary

A total of 40320 data points were analyzed per air temperature sensor and per temperature trial, amounting to 645,120 total data points. **Table 2** shows the descriptive statistics for the raw data with NA count for all four sensors per temperature trial. The proposed alternative decomposition method achieved similar success at identifying uncertainty occurrences and deviations as standard additive decomposition function as shown on **Table 3**.

**Table 2:** Summaries of raw air temperature data and number of missing values per sensor and per temperature trial.

Sensor	Trial	Raw Data					
		Mean	Median	Stan. Dev.	Min	Max	NA
		(°C)	(°C)	(°C)	(°C)	(°C)	count
LC_Reference	T1	22.677	25.16	3.466	15.75	28.7	19649
	T2	26.097	29.84	5.251	16.33	31.71	13837
	T3	23.8	27.16	4.56	15.36	30.87	17525
	T4	22.202	23.72	2.393	16.2	26.1	666
LC_Canopy_1	T1	23.668	26.21	3.46	17.43	28.95	19649
	T2	27.756	31.48	5.279	19.07	33.49	13837
	T3	24.787	28.21	4.557	17.09	31.19	17525
	T4	24.423	26.16	2.578	20.1	26.75	666
LC_Canopy_2	T1	22.534	24.93	3.503	17.3	27.05	19650
	T2	25.734	29.3	5.09	17.37	31.15	13837
	T3	23.665	26.76	4.379	17.47	29.03	17525
	T4	21.729	23.16	2.328	17.08	25.31	666
Reference	T1	23.547	25.5	3.26	16.4	28.8	1182
	T2	26.788	30.2	5.068	16.85	31.8	0
	T3	24.925	27.5	4.266	15.9	30.88	0
	T4	22.515	24.05	2.373	17	26	0

Raw data refers to the unaltered data collected from each sensor. NA = count of missing values. The LC_Canopy_1, and LC_Canopy_2 (SHT30) sensors were placed at the two different plant canopies, while the LC_Reference and Reference (APCEM2) sensors were adjacent to each other and away from the plant canopies to monitor overall environmental conditions.

**Table 3:** Comparison of residual data mean, standard deviation, and number of outliers identified by the standard and alternative decomposition methods per sensor and per temperature trial.

Sensor	Trial	Standard Additive Decomposition			Alternative Decomposition		
		Mean	Stan. Dev.	Outlier	Mean	Stan. Dev.	Outlier
LC_Reference	T1	-0.012	0.516	105	-0.005	0.394	27
	T2	0.007	1.399	1219	0.105	1.516	1286
	T3	0.001	0.446	47	-0.025	0.509	51
	T4	0.003	0.485	85	0.01	0.527	183
LC_Canopy_1	T1	-0.013	0.469	81	-0.011	0.33	0
	T2	0.005	1.341	1191	0.086	1.448	1242
	T3	0	0.396	10	-0.029	0.459	4
	T4	0.003	0.36	20	0	0.391	66
LC_Canopy_2	T1	-0.017	0.487	80	-0.026	0.377	2
	T2	0.005	1.337	1197	0.091	1.446	1272
	T3	0.001	0.429	0	-0.043	0.499	15
	T4	0.001	0.439	47	0.012	0.482	142
Reference	T1	-0.003	0.432	153	0.06	0.204	0
	T2	0.009	0.496	18	0.066	0.779	593
	T3	0.002	0.188	0	0.064	0.367	0
	T4	0	0.056	0	-0.016	0.068	0

The LC_Canopy_1, and LC_Canopy_2 (SHT30) sensors were placed at the two different plant canopies, while the LC_Reference and Reference (APCEM2) sensors were adjacent to each other and away from the plant canopies to monitor overall environmental conditions. Standard additive decomposition was performed with the base R statistics package decompose() function, and subtracted the coalesced data from the identified seasonal and trend data. Alternative decomposition method subtracted the coalesced data from the surrogate data. Outlier = count of residual values with z scores greater than or equal to 3.29 (|z| ≥ 3.29).

### 3.2 Residual distribution and curve fitting

**Figure 4:**
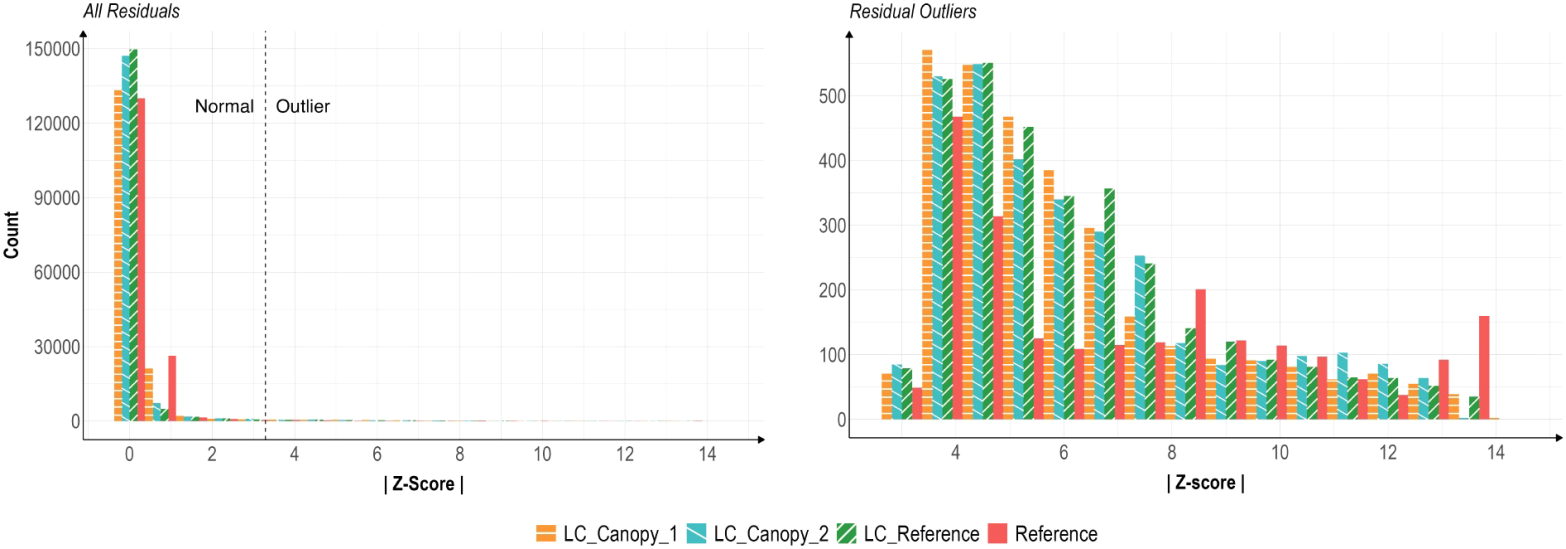
Histograms of air temperature residual z-scores of all values (left) and of outlier-only (|z| > 3.29) values (right). Residuals were calculated via alternative decomposition method with data from sensors during all four temperature trials. The LC_Canopy_1, and LC_Canopy_2 (SHT30) sensors were placed at the two different plant canopies, while LC_Reference and Reference (APCEM2) sensors were placed next to each other away from the plant canopies to monitor overall environmental conditions.

The distributions of z-scores of all low-cost (LC) sensors was similar across the four trials, with differences seen only in the Reference sensor data (**Figure 4**).

A constant bias or trend in the Reference sensor data away from the LC sensor data could have been due to differences in sensor hardware, however these types of discrepancies were not clear in the data. The Reference and LC_Reference sensor data sets should also have had similar disparities in quality that differed them from the remaining LC sensors, but this too was not reflected in the data. From these results, we concluded that the disparities in data quality must have been due to any one or a combination of the types of uncertainties listed in **Table 1**, though which is unclear. The differences in residual distribution also suggests that comparing data readings across sensor types may not be an effective means of studying the differences in data quality. Due to these data discrepancies, the Reference data was excluded from further curve-fitting analyses as it would affect the accuracy of our statistical models.

Goodness-of-fit tests were performed on the combined residuals of all LC sensor data across the four trials to identify the best fit distribution model (**Table 4**). The test results revealed that the log-normal distribution model provided the best fit for the data, with parameters estimated as meanlog = 1.7300694 and sdlog = 0.3805325. The log-normal model achieved a log-likelihood of -20379.76, an AIC of 40763.52, and a BIC of 40777.82, indicating a better fit when compared to the other distribution models.

**Table 4:** Maximum goodness-of-fit estimations for multiple distribution curves over the combined residual distributions of low-cost sensor data.

Distribution	Parameter Estimate		Loglikelihood	AIC	BIC
Normal	mean: 5.765050	sd: 2.086998	-22251.02	44506.04	44520.34
Log-normal	meanlog: 1.7300694	sdlog: 0.3805325	-20379.76	40763.52	40777.82
Gamma	shape: 7.291360	rate: 1.224135	-20723.16	41450.32	41464.62
Weibull	shape: 3.027225	scale: 6.509577	-22045.32	44094.64	44108.94
Logistic	location: 5.753100	scale: 1.266623	-21677.08	43358.17	43372.47
Cauchy	location: 5.686519	scale: 1.295663	-22690.9	45385.8	45400.1

Results generated by the fitdist() function with respective distribution types, and method “mge” from the “fitdistrplus” library.

**Figure 5:**
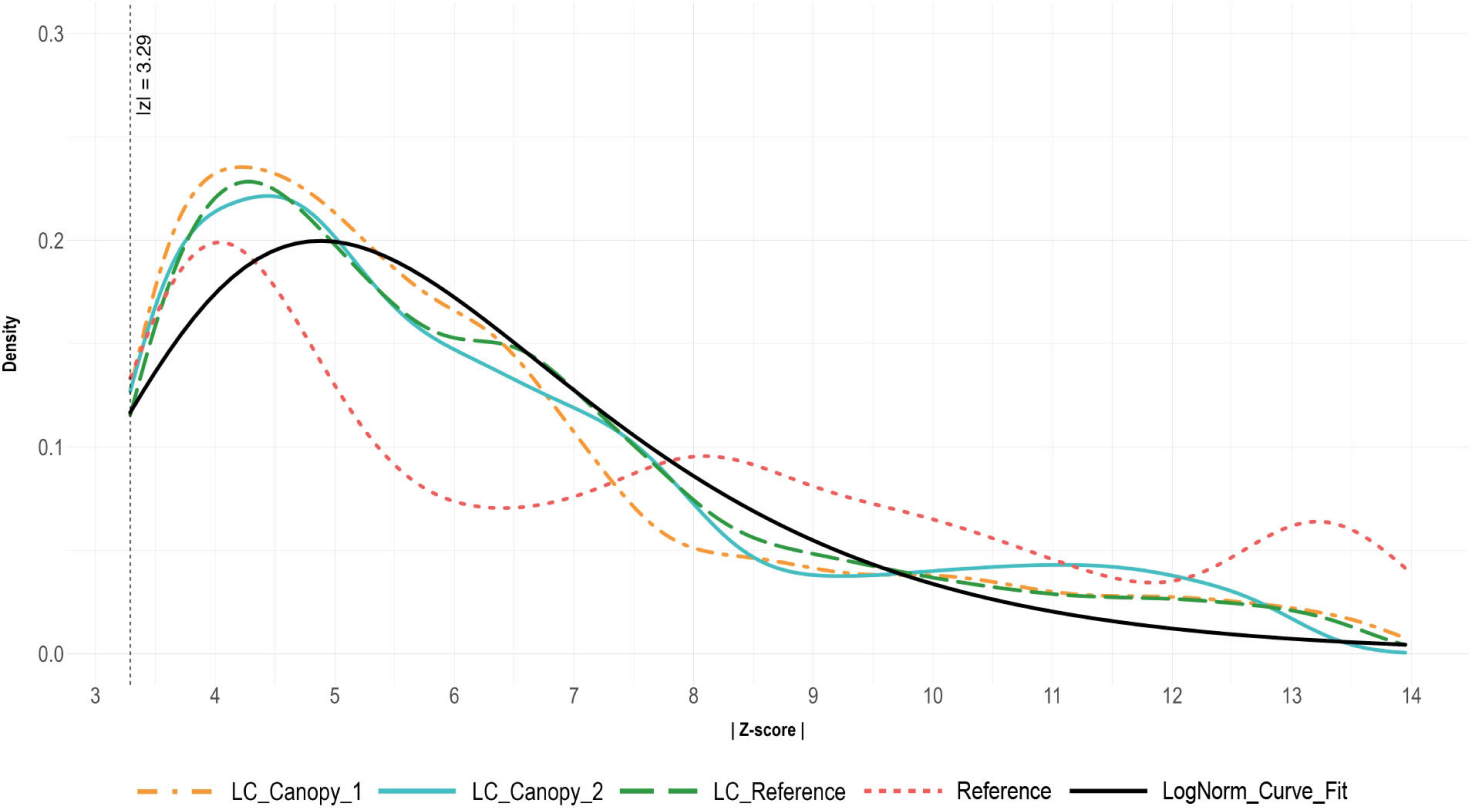
Density plots of the absolute value of residual z-scores (|Z-score|) for outlier data (|z| > 3.29) only. Residuals were calculated via alternative decomposition method with data from sensor during all four temperature trials. The LC_Canopy_1, and LC_Canopy_2 (SHT30) sensors were placed at the two different plant canopies, while LC_Reference and Reference (APCEM2) sensors were placed next to each other away from the plant canopies to monitor overall environmental conditions.

**Figure 4** presents two histograms of residual values, the first shows the distribution of all residuals, while the second shows the distribution of outlier-only residuals with |z| > 3.29. The distribution of all LC sensor residual values is extremely skewed left, with a long thin tail. For the outlier-only values, the LC sensors demonstrate a spread similar to a gamma or log-normal distributions, while the Reference sensor data distribution appears to be slightly bimodal. Density plots of the outlier-only data for each sensor, and a curve representing the best-fit log-normal curve for the LC sensor data can be seen in **Figure 5**.

### 3.3 Regression analysis

**Figure 6:**
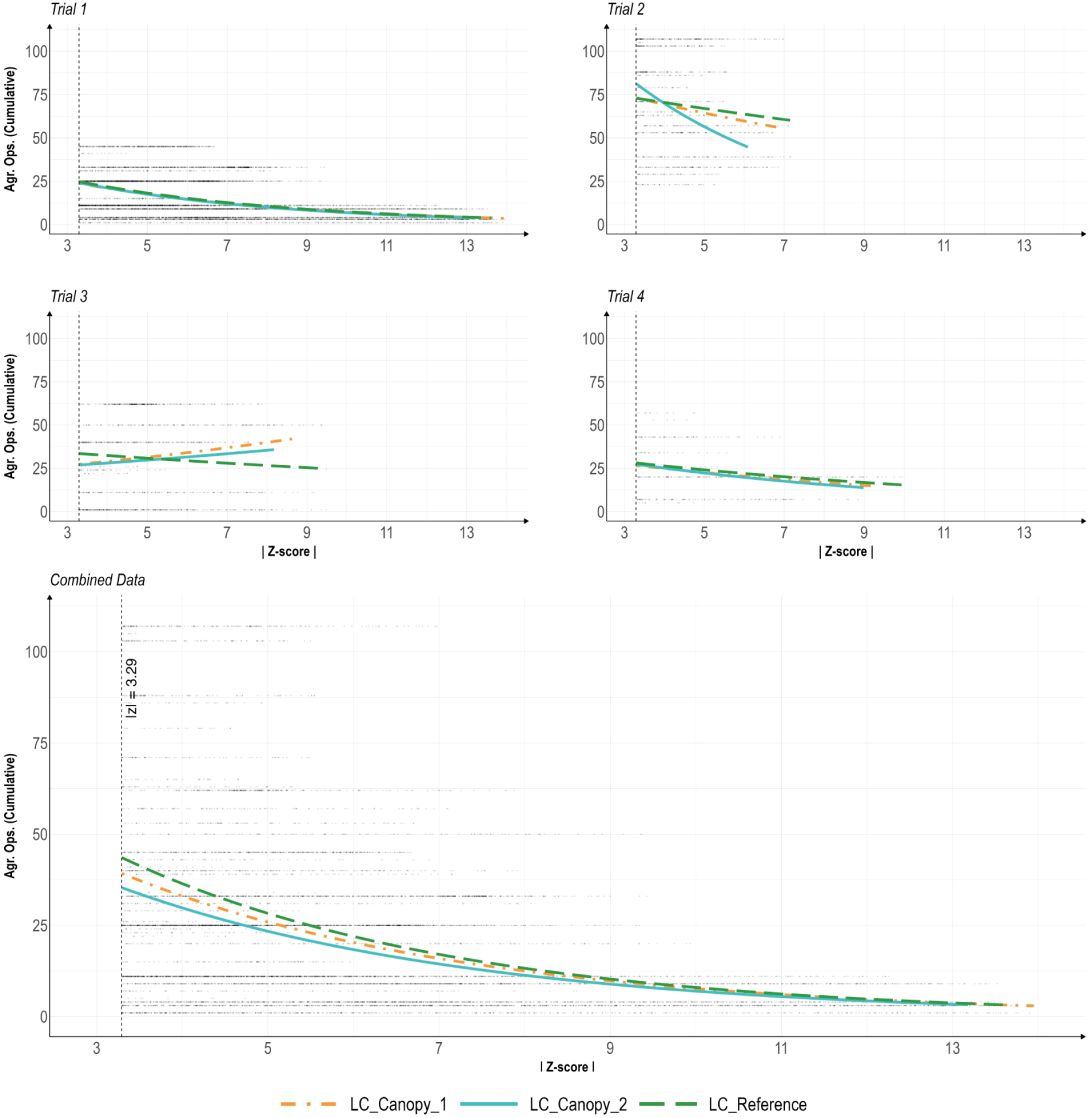
Regression curves for agricultural operations (Agr. Ops) and the absolute value of residual z-scores (|Z-score|) for outlier data (|z| > 3.29) only, per trail and combined trials. Analysis includes residual data calculated with alternative decomposition method from each sensor throughout all four temperature trials. The LC_Canopy_1, and LC_Canopy_2 (SHT30) sensors were placed at the two different plant canopies, while the LC_Reference sensor was placed away from the plant canopies to monitor overall environmental conditions.

Regression analysis for a log-normal distribution was performed on the data of the individual LC sensors per trial and on a combination of all trial data, with individual analyses performed per sensor (**Figure 6**). The regression curve of LC_Canopy_1 shows some minor positive correlation in trial 3, but the remaining regression curves for trial 1-4 show slight negative correlations that reduces as z-scores increase. Similarly, the analysis of the combined data showed a slightly negative correlation at lower z-score values that reduced at higher z-score values. The results of the regression analysis show a strong inverse relationship between Agr.Ops and the residual z-scores (**Table 5**). Among the three sensors, LC_Canopy_1 had a significant negative interaction effect with Agr.Ops, but neither of the other two LC sensors showed any significant effect. These results suggest that Agr.Ops could be a contributing factor to the outlier presence in the data over time, which partially proves our initial hypothesis of the effect of Agr.Ops on data quality. But the validity of this relationship need to be judged case by case.

**Table 5:** Summary of regression analysis results for combined goodness-of-fit estimations for multiple distribution curves over the combined residual distributions of low-cost sensor data. Results generated by the fitdist() function with respective distribution types, and method “mge” from the “fitdistrplus” library. .

Coefficient	Estimate	Std. Error	t value	Pr(>|t|)	Significance
(Intercept)	1.8944395	0.0047165	401.661	< 2e-16	***
Agr_Ops	-0.0061776	0.0001468	-42.088	< 2e-16	***
LC_Canopy_1	-0.0160957	0.0094928	-1.696	0.08998	.
LC_Canopy_2	0.0120864	0.0094614	1.277	0.20146	
LC_Reference	0.0078452	0.0093914	0.835	0.40352	
Agr_Ops: LC_Canopy_1	0.0002833	0.0002929	0.967	0.33341	
Agr_Ops: LC_Canopy_2	-0.0009709	0.0003189	-3.045	0.00233	**
Agr_Ops: LC_Reference	0.0003582	0.0002769	1.293	0.19587	

## 4 Discussion

This study shows a new pathway of assessing the stability of a controlled environment agriculture system by investigating the status of its data quality (e.g., outliers). From the perspective of system managers, the overall stability of the operating environment is related to the risk control for avoiding system failure, plant stress, management difficulties.

### 4.1 Outlier detection and system stability

Using this alternate decomposition method could provide insight into the functionality of the indoor farming system by identifying shifts away from the “ideal” operation represented by the surrogate data. These outlier data could be indicative of sensor noise, drift or linearity errors, or one of the many uncertainties that can affect these systems (**Table 1**), since all of these could be perceived as significant shifts from the surrogate data. However, since this method cannot differentiate between these uncertainties well, it may require smaller alpha values (α < 0.001) to assist in identifying only the most extreme values as true outliers.

When associating outliers with uncertainties, the magnitude of the z-score is a critical measure of these uncertainties’ significance. A large z-score indicates severe deviations from the norm, implying potential system instability. In our study, Trail 1 exhibited the largest z-score (>13), indicating significant vulnerabilities and a high risk of system failure, likely due to initial setup conditions requiring fine-tuning. The other trails, with maximum z-scores around 7 or 9, experienced less severe uncertainties, suggesting better stability. Trail 2, with a maximum z-score around 7, showed relative stability compared to Trail 1.

The impact of Agr.Ops on outliers can also be informed through regression analysis. Ideally, if Agr.Ops influenced system stability, a non-negative regression trend would be observed. However, all trails, except Trail 3, demonstrated a negative trend, indicating that the severeness of uncertainties did not increase over time. In Trail 3, a positive trend was observed for LC_Canopy_1 and LC_Canopy_2, suggesting that Agr.Ops impacted these specific readings but not the overall system stability as indicated by the LC_Reference data. This could be due to poor air circulation and high operating temperatures (28°C), warranting further investigation.

In Trails 1, 2, and 4, the initial growth stages of lettuce were associated with significant uncertainties, which could be due to the young plants being unable to stabilize their microclimate effectively (Hassanien Emam Hassanien and Ming, 2017). Over time, as plants grew and the canopy area increased, the system became more resistant to Agr.Ops influences, resulting in smaller z-scores and fewer outliers. Trail 2, with a notably steeper negative slope, demonstrated higher resistance to Agr.Ops influences compared to Trails 1 and 4. Additionally, Trail 4 had fewer missing data points, indicating better data reliability, likely due to fewer power outages.

Overall, while Trail 3 showed increased instability over time due to Agr.Ops, Trails 1, 2, and 4 generally became more stable as plants grew. Trail 2 was the most stable, with the steepest negative slope and narrowest z-score range. For VF operations, achieving system stability requires balancing small outlier z-scores, ensuring outlier occurrences are independent of Agr.Ops, and maintaining reliable data collection.

### 4.2 Implications of uncertainties

Other detected outliers however show seemingly no correlation with agricultural operations, but could be attributed to other uncertainties or fluctuations in system performance. Some outliers occurred at the scheduled day/night period temperature shifts and could be caused by slight delays in the AC unit activation for cooling/heating the system since a temporal delay could show up as a significant enough difference in the data to be identified as an outlier. Other outliers may be indicative of changes in the dynamics of an indoor farming system and could be attributed to a number of different uncertainties that if identified would help to better understand the overall system health. For example, as plants grow larger they will absorb a greater amount of radiation from a light source, transpiring more as more photosynthesis reactions occur. The changes that occur in an indoor farm during plant growth and development can be non-linear and could even alter the rate of energy consumption and the amount of time required for the system to reach equilibrium.

When managing an indoor plant growing system, certain agricultural operations may only occur at specific stages of plant growth (Ex: For lettuce, transplanting at week 1, harvesting at week 5), and the effects of these specific operations on the system data can be easily tracked and understood. Similarly, the regular day/night temperature fluctuations used for environmental control become a part of the identified daily periodicity, and should not shift greatly over time as long as the components used to control these are in good condition. Thus, if the environmental conditions begin to shift away from the regular periodicity in the data, these shifts can be attributed to a change in the system operation or the plant growth and development and can be flagged for investigation. Whether the changes are caused by agricultural operation duration, machinery or sensor failure, biological factors, or some other uncertainty, they can be identified if compared to the surrogate data. A large indoor plant growing system (such as a commercially operating plant factory with plants at varied growth stages) could generate a large amount of sensor data that would consequently make for high quality surrogate data extraction and decomposition. These larger systems require fine-tuned control for multiple components, and using this method could shed light on the operational stability of the system as a whole, or even the status of individual components. For example, artificial light sources could have their temperature monitored to identify a gradual decay in their luminous efficacy if the amount of heat generated increases over time, since this would mean that more energy is being lost as heat rather than being converted to light.

Stacking the daily data in this way makes the direct correlations more difficult to identify, but can better show the frequency of outliers based on daily operation times. Looking more closely at the outliers identified during day/night temperature changes, these outliers were likely identified due to being temporally shifted from the surrogate data, and since very low intensity or no agricultural operations were performed during those times, are unlikely to be caused by an operational uncertainty. Instead, these changes could indicate that the air temperature sensor for the AC unit is decaying over time and cooling/heating less effectively, or, the increasing mass of the system caused by plant growth is requiring more time to cool/heat effectively. In either case, a potential dynamic of the system is being identified to be investigated so that we can better understand why the system is fluctuating as it is.

### 4.3 Future directions

Existing studies on CEA regarding system stability mostly focus on control based on system feedback (Ngo et al., 2020), variable selection (De Goede et al., 2013), or understanding the system’s response to changes (Urruty et al., 2016). However, the assessment of overall system stability using environmental or other such monitoring data in terms of statistical performance is scarce. This study fills this gap and provides a procedure for others to follow in addressing problems, such as missing data, outlier detection, and correlating Agr.Ops with outliers, which can be used to indicate system stability.

Understanding the sources and impacts of these uncertainties is crucial for improving system reliability. Future research could focus on developing more sophisticated models to differentiate between types of uncertainties. Additionally, implementing predictive maintenance strategies based on sensor data could help preemptively address issues like sensor degradation or system inefficiencies.

Utilizing surrogate data for outlier detection proved to be an effective method for identifying deviations from ideal operation conditions. This approach could be further enhanced by refining the surrogate data generation process. For example, incorporating more complex models that account for plant growth stages and their impact on environmental conditions could improve accuracy. Exploring machine learning techniques to create adaptive surrogate data models that evolve with system changes might also be a valuable avenue for future research.

The observed discrepancies in the Reference sensor data compared to the LC sensors suggest that sensor hardware differences can impact data quality. This variability underscores the need for careful sensor selection and calibration in indoor farming systems. Future studies could compare a broader range of sensors under different environmental conditions to establish guidelines for optimal sensor use in various farming scenarios.

The frequent occurrence of missing data in some systems, such as Trail 1,2,3, highlights the importance of data redundancy. Implementing multiple sensors and cross-verifying their readings can mitigate the impact of data gaps and enhance overall data quality. Exploring sensor network designs that incorporate redundancy and failover mechanisms could be a promising direction for future research.

## 5 Conclusion

This study analyzed air temperature data from indoor farming systems using an alternative decomposition method based on surrogate data as a representation of the periodicity of environmental sensor data to assess system performance and stability. Data from multiple sensors was used to compare a standard decomposition with the alternative method, and it was found that the alternative method was effective in detecting outliers. The outlier detection method used here can be adapted to any sensor data that displays regular seasonality in a controlled environment setting. The simplicity and low computational requirement of this method makes it effective for IoT infrastructure, and can be utilized with offline, online, static, dynamic, logged data or real-time data collection. However, this method is reliant on the quantity and accuracy of data collected and is thus impacted by sensor data quality and quantity sampled, which has a simple but potentially costly remedy of using larger quantities of sensors.

Regression analysis showed an inverse relationship between cumulative agriculture operations and the intensity of deviated condition (residual z-scores). The log-normal distribution model best characterized the residuals, highlighting the importance of monitoring uncertainties. Systems with less impact of agriculture operations (either few outliers or fading severeness overtime) and lower z-scores of outliers were deemed more stable. These findings underscore the importance of managing uncertainties in indoor farming systems to enhance reliability and efficiency, with the alternative decomposition method offering a promising approach for improved system monitoring. However, the data collected and studied in this experiment was of relatively short duration, and used few sensors. Further studies on the reliability of the proposed surrogate data collection and alternative decomposition method as data diagnostic tools should be performed. Future research should further validate these methods in larger commercial operations.

## 6 Appendix

**Figure 7:**
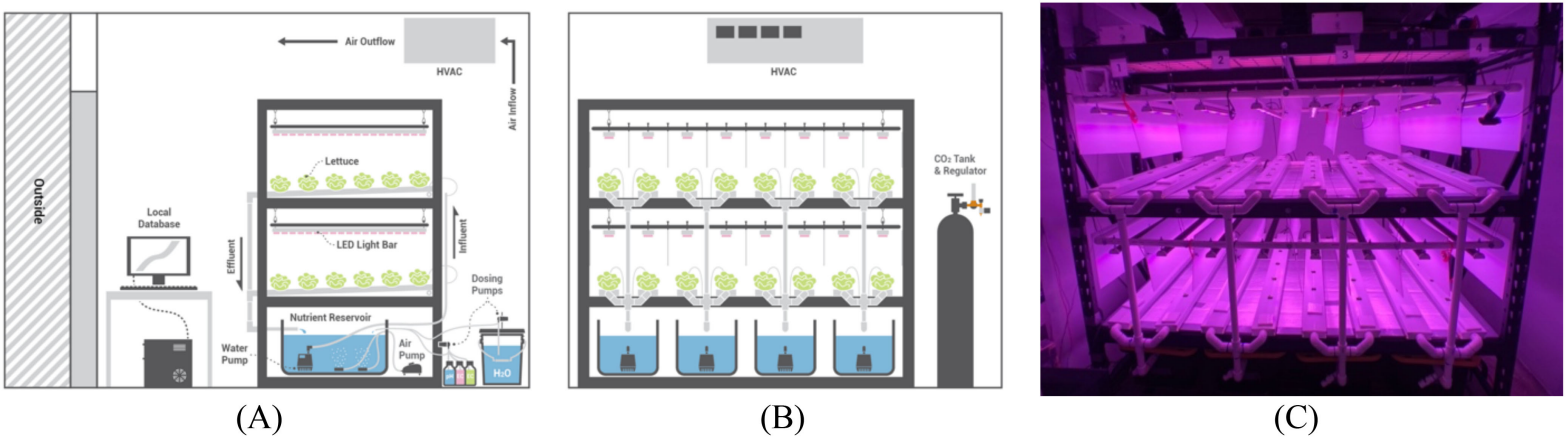
Indoor farming system diagram where lettuce was grown with a fully automated hydroponic system: **(A)** side elevation; **(B)** front elevation; **(C)** image of actual growth chamber used before the transplanting of lettuce seedlings into the channels.

An environmental growth chamber (Environmental Growth Chambers, TC2, Chagrin Falls, Ohio) of 2.74 m × 2.74 m × 2.39 m (width × depth × height) contained the indoor farm system used in for this experiment. Temperature, relative humidity (RH), and airflow in this chamber were controlled by the heating, ventilation, and air-conditioning (HVAC) system with continuous fan operation. Two 2.13 m × 0.61 m × 2.13 m (width × depth × height) shelving units placed front to back created the shelf surfaces on which the hydroponic systems were held (**Figure 7**). A solenoid valve regulator was used to enrich the CO_2_ in the chamber to a constant 800 mg/L. A day/night interval of 16.5/7.5 h (Brechner and Both, 2013; Caplan, 2018) was used for both lighting and temperature control. RH was controlled by the chamber HVAC system and was set to a maximum of 60% RH with no minimum. Vertical white plastic reflectors were placed between the NFT channels provide uniform lighting conditions to all plants in the system. Four temperature trials were conducted where lettuce was grown for 7 days in an Ebb & Flood system, and then transplanted into the NFT channels at day 7 until harvested on day 35. Agricultural operations consisted of opening/closing the chamber door, gathering data, recalibrating sensors, refilling nutrient solution reservoirs, harvesting lettuce, and general maintenance tasks within the chamber.

### 6.1 Data collection system

Six microcontroller boards were used to separate the LC sensors to provide adequate power for each sensor and ensure high interval data collection. Five MEGA2560 boards (Elegoo, MEGA 2560, Shenzen, China) were used for the environmental monitoring and control, and one ESP32 (Espressif Systems, ESP32-S2-Saola-1, Shanghai, China) board was used for agricultural operations monitoring. All six boards were connected to a single computer tower that acted as a data logger by logging data online to a MySQL database, and locally as a.txt file with python scripts. All data was timestamped after being collected in the Python serial monitor (RRID:SCR_008394) to ensure the date-time was accurate and experienced no drift.

**Figure 8:**
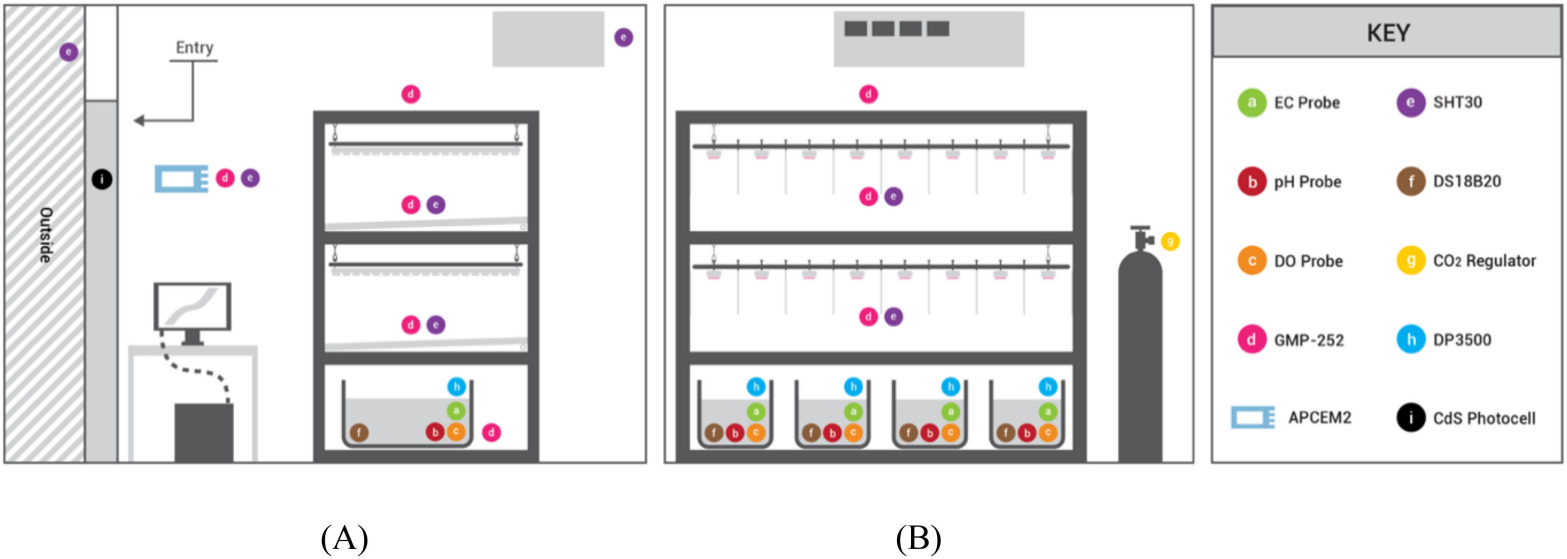
Diagram of sensor placement in the growth chamber: **(A)** side elevation; **(B)** front elevation.

Sensor data was collected for the full five weeks of each of the four trials. The first week of each trial was identical to have uniform germination conditions and seedling growth rates. The seedlings would be exposed to experimental conditions from week 2-5 to test the changes in growth and development of the plants at the varied air temperature setpoints. LC sensor data was collected at 10 sec intervals and included air temperature, relative humidity (RH), CO_2_ concentration, electrical conductivity (EC), pH, dissolved oxygen (DO), root-zone temperature, the dosing quantity for nutrient/water/acid/base, and agricultural operations performed. This data collection resulted in a 302,400 data points for each of the 50 parameters measured per trial. The reference system collected data at 5 second intervals amounting to 604,800 data points per parameter per trial. Unique parameters were averaged per minute, and these averaged data sets were used for data analysis and visualization. Each of the four trials had a different air temperature set point (24, 26, 28, 30°C), so unique data sets were averaged per trial. Sensors were placed at varying locations in the chamber as can be seen in **Figure 8**. Air temperature, RH, and CO_2_ data were collected at the two plant canopy levels to ensure the operation of the system matched the needs of the plants being grown and to measure the environmental uniformity. The reference sensor was placed away from the growing system to monitor the overall chamber conditions. LC air temperature, humidity and CO_2_ sensors were placed next to the reference sensor to compare data gathered in identical conditions. Each nutrient solution reservoir was monitored for pH, EC, DO, temperature, and a float sensor to maintain desired conditions.

### 6.2 Sensor matrix

**Figure 9:**
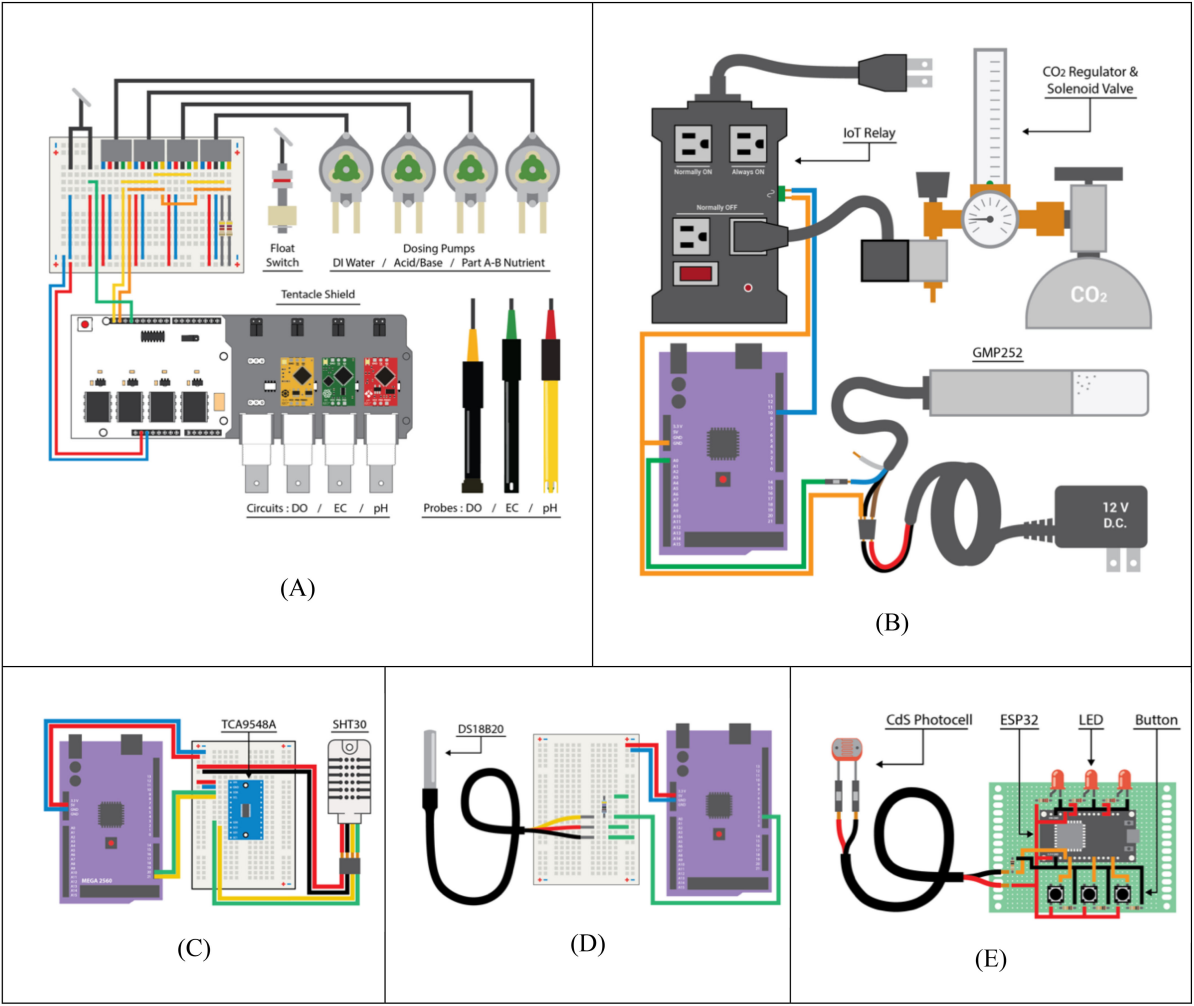
Sensor wiring diagrams with Arduino and bread boards for data collection. **(A)** Nutrient solution system; **(B)** CO_2_ concentration; **(C)** temperature and humidity; **(D)** nutrient solution temperature; **(E)** agricultural operations.

A reference data set was collected from a standalone sensor and data logger (Hydrofarm, APCEM2, Petaluma, CA), which collected air temperature, CO_2_ concentration, and RH, and recorded it locally into a microSD card. The water reservoir volumes were monitored with float switches (Anndason, DP3500, Shenzen, China) (**Figure 9A**). EC, pH, and water levels were maintained via automatic dosing by peristaltic pumps (Atlas Scientific, EZOTM-PMP, Long Island City, NY) based off of their respective sensor readings. CO_2_ concentration was monitored with five infra-red CO_2_ (Vaisala, GMP252, Vantaa, Finland) sensor probes, and controlled by a CO_2_ tank regulator and solenoid valve connected to a relay (**Figure 9B**). Five air temperature and relative humidity sensors (Adafruit, SHT30, New York, NY) were placed at different elevations and locations (both plant canopies, HVAC intake, beside the reference sensor, outside the chamber) to monitor the conditions inside and outside the chamber. A multiplexer (Adafruit, TCA9548A, New York, NY) switched between the five sensors of identical I^2^C address (**Figure 9C**). The nutrient solution temperatures were monitored with waterproof type T thermocouples (Aideepen, DS18B20, Shenzen, China) calibrated with water at 100°C and 0°C (**Figure 9D**). The date/time data of agricultural operations performed by entering the chamber were recorded using a photoresistor (Adafruit, CdS photocell, New York, NY) to log the chamber door open or closed state, and with manual button switches to record the presence of up to three people in the chamber at a time (**Figure 9E**). Atlas Scientific sensor probes and their corresponding EZO™ embedded circuits were also used to monitor the nutrient solution. These sensor arrays included pH (Atlas Scientific, #ENV-40-pH, Long Island City, NY), electrical conductivity (Atlas Scientific, #ENV-40-EC-K1.0, Long Island City, NY), and dissolved oxygen (Atlas Scientific, #ENV-40-DOX, Long Island City, NY). The EZO™ circuits were mounted onto a tentacle shield (Whitebox, T1.16, Schlatt TG, Switzerland) that provided circuit breakouts, electrical isolation, and probe connection ports. Cumulative electrical consumption was measured with current meters (CrocSee, CRS-022B, Shenzen, China).

### 6.3 Data management

How data is collected, managed, and stored can play a significant role in the integrity of any system relying on sensor based automation and control. An indoor farming system that collects data at high frequency needs to be able to store large data sets in a reliable and accessible manner to ensure the system is operating at optimal capacity, and is being controlled within the desired environmental parameters. Analysis of collected data can reveal system requirements, uncertainties experienced, errors in data, and other such operational insights.

#### 6.3.1 Database design

**Figure 10:**
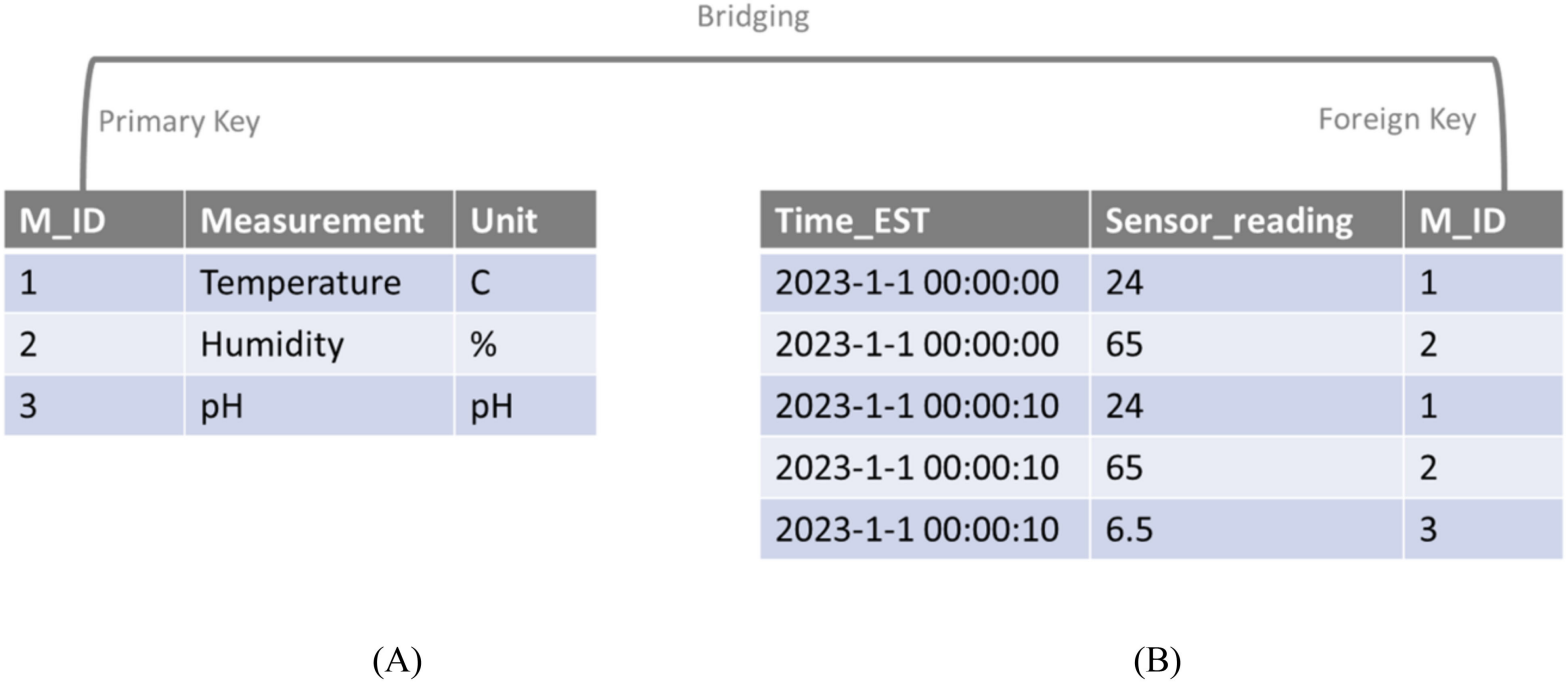
Structure of measurement table **(A)** and sensor_data table **(B)**. M_ID is the key in bridging two tables.

The data collection system requires: 1) real-time data storage and 2) adaptation of adding/removing devices. The design of the database should fulfill these requirements. Specifically, the database is constructed with MySQL service, which provides functionality for storing real-time data. Data is uploaded from Arduino boards via the API of MySQL and dumped into a table (sensor_data) recording the time stamps, values and M_ID (a unique ID assigned to each sensor) (**Figure 10**). To address the issue of adding, removing, and replacing sensors, we established and managed a measurement table serving as the reference of sensor devices. This table includes the unit, type of measurement, and the identity of each sensor. The identities of sensors, corresponding to the M_IDs in sensor_data, are set as the primary key (a connection index information to connect multiple tables in a regional database, such as MySQL) for querying the senor-specific information in the database. The bridging built on M_IDs between two tables allows us to manage the database more efficiently. For example, once a reading is recorded in the sensor_data table, its corresponding measurement and unit can be traced down in the measurement table by the M_ID. Similarly, when a new sensor is connected to the Arduino board, a row with a new M_ID representing this sensor can be appended to the measurement table. The data collected from new sensor is added to the sensor_data table with its corresponding M_ID. With this procedure, a static database structure can be used to facilitating a dynamic CEA system.

#### 6.3.2 Backup file system

The backup file system is a crucial component for data security, and works by aggregating all data within the system into a comprehensive data stream and then automatically writing this data stream into a.txt file stored on the local computer hard-drive once per day. This involved scanning and compiling each unique data stream to ensure a complete data flow. By transforming these data sets into a sequential stream, it became easier to manage and was optimized for storage space requirements. A.txt file format was used to back-up the data locally since this file type is a simple, efficient format renowned for its compatibility and ease of use. This process was designed with data integrity in mind, allowing for the original data to be re-created seamlessly from the locally stored data. The backup file system’s ability to convert and store data in this way provides an effective safeguard against potential data loss incidents from a system crash, human error, or other unforeseen circumstances. Thus, it serves as a key player in maintaining data safety and stability. However, there many other uncertainties that can affect data collection in an indoor farming system, and can also result in data loss, corruption, or other such errors. For our experiment, the expected uncertainties are listed in **Table 1**. Many methods exist to identify and remedy these uncertainties, from modifying the system design, to data processing, but no method is perfect.

## Data Availability

The datasets presented in this study can be found in online repositories. The names of the repository/repositories and accession number(s) can be found below: https://github.com/JeanPompeo/Hydroponic_Control_System_Chamber_1.git.
